# Evaluation of a co-designed Parkinson’s awareness audio podcast for undergraduate nursing students in Northern Ireland

**DOI:** 10.1186/s12912-023-01544-x

**Published:** 2023-10-09

**Authors:** Sophie Crooks, Patrick Stark, Susan Carlisle, Johanna McMullan, Shannon Copeland, Wai Yee Amy Wong, David Blake, Elaine Lyons, Nuala Campbell, Gillian Carter, Christine Brown Wilson, Gary Mitchell

**Affiliations:** 1 School of Nursing and Midwifery, Queen’s University Belfast, Belfast, Northern Ireland; 2 University of East Anglia, Norwich Medical School, Norwich, England; 3 Parkinson’s UK, Belfast, Northern Ireland

**Keywords:** Parkinson’s Disease, Podcast, Education, Nursing student, Undergraduate, Asynchronous, Co-Design, Mixed methods

## Abstract

**Background:**

Parkinson’s Disease (PD) is a common neurological condition that often causes stiffness, tremor and slow movement. People living with PD are likely to encounter nursing students throughout their journey from pre-diagnosis to death. Despite this, there is a paucity of evidence about current practice in PD education amongst nursing students. The present study provides an evaluation of a co-designed Parkinson’s Awareness audio podcast amongst nursing students in Northern Ireland.

**Methods:**

Following co-design of an audio podcast about PD, a mixed methods evaluation was carried out. 332 student nurses completed pre-/post-test questionnaires about their knowledge and perceptions of PD before and after listening to the audio podcast. Further to this, 35 student nurses participated in focus-group interviews six months following listening to explore how the podcast influenced practice.

**Results:**

Student nurses posted a mean score of 52% before listening to the audio podcast. This mean increased to 80% post-test. These findings were statistically significant (p < 0.001), demonstrating significant increases in PD awareness after listening. Findings from the focus groups suggested that the audio podcast improved empathy and practice towards people with PD. The findings also suggested that students perceived audio podcasts to be a good way to learn about PD.

**Conclusion:**

Provision of a co-designed audio podcast about PD has the potential to improve student nurse knowledge and practice related to PD as evidenced in this study.

**Supplementary Information:**

The online version contains supplementary material available at 10.1186/s12912-023-01544-x.

## Introduction

Parkinson’s Disease (PD) is the second most common neuro-degenerative disease in the world, caused by reduced dopamine levels in the brain [1]. It is characterised by both motor and non-motor symptoms including tremor, unsteady gait, rigidity, slurred speech and sleep disorders [2, 3]. The global prevalence of the disease is over 6 million people, increasing by 2.5 times within the past 30 years [4]. It is estimated by 2025, the global burden of PD will increase by 23.2% [5]. The cause of this increase in PD has been related to population growth and ageing, genetic predisposition, lifestyle changes and environmental pollution [6]. Alongside this, improved diagnostic methods and increased awareness of the disease has been attributed to a rise in incidence trends [7].

Research has highlighted the lack of awareness of PD, in particular the lack of knowledge amongst healthcare professionals [8–10]. With increasing prevalence of PD, it is expected that more people will require hospital care, therefore it is imperative that healthcare professionals are provided with educational resources to improve awareness and knowledge of the disease [3, 10–12]. Greater education also helps those working in healthcare provide better quality, person-centred care to patients [13]. Educating nursing students is particularly important to ensure they are provided with resources to enable them to provide holistic care to people living with the disease [14]. Despite this, there appears to be a lack of empirical evidence that describes and evaluates nursing student education for PD. This is surprising given the important role student nurses have in providing care to patients in both the clinical and community setting [15].

Technological advances such as computing, internet and mobile phones have formed the direction of continuous education, enabling educators to design and deliver innovative and interactive content that is easily accessible [16, 17]. Podcasting is a tool that has been recently utilised within nursing education due to the ease which content can be created and shared online [16]. Evidence shows they can be a convenient, effective and an engaging way to disseminate knowledge [18–21]. Recent advancements in medical education have seen the rapid adoption of podcasts as versatile and flexible tools for learning and there is a growing acceptance of healthcare professions education podcasts across different training levels [22–24]. Learners appreciate the convenience and just-in-time learning offered by podcasts, finding them efficient and enjoyable for staying up to date [22]. Moreover, the appeal of podcasts extends beyond conventional educational methods, as they provide exposure to international expertise and create a low-stress learning atmosphere [23]. This aligns with the principles of andragogy, empowering adult learners to plan their content, making podcasts a valuable resource, particularly for advanced learners [22].

Notably, the podcasting landscape in health education is diverse, with a range of formats and affiliations. Research by Trad and colleagues revealed that the 100 most popular medical podcasts in the United States exhibit a variety of didactic methods, including those ranked highly on Bloom’s taxonomy. A significant portion of these podcasts is intended for physician education, covering various levels of medical learners [24]. Furthermore, many of these podcasts offer references, peer review processes, and even continuing medical education (CME) credits, enhancing their credibility and educational value [24]. These findings underscore the need for ongoing research and integration of podcasts into healthcare education. While there are gaps in understanding the optimal use and effectiveness of podcasts, their potential to reach a broad audience, including healthcare students, newly qualified professionals, and practicing clinicians, is evident. As healthcare education continues to evolve, podcasts offer a powerful medium to disseminate innovative practices, bridge knowledge gaps, and empower learners across diverse settings such as primary, secondary and tertiary care [22–24]. The dynamic nature of podcasts, along with their ability to foster positive learning environments and share cultural competencies, positions them as essential tools for modern clinical education [23, 24].

Building on the emerging evidence of podcasts as valuable educational tools in health professions education, our study extends the evidence-base to nursing education. Recognising the potential benefits highlighted in previous research, this study sought to.

Evaluate the effectiveness of a co-designed Parkinson’s awareness podcast for undergraduate nursing students in Northern Ireland. The integration of a Parkinson’s awareness podcast is a pedagogically valuable strategy for enhancing undergraduate nursing education in the United Kingdom. This educational approach is congruent with the Nursing and Midwifery Council’s (NMC) Standards of Proficiency for Registered Nurses (https://www.nmc.org.uk/globalassets/sitedocuments/education-standards/future-nurse-proficiencies.pdf), particularly with reference to Annex B, which emphasises essential nursing procedures encompassing assessment, planning, and the delivery of person-centred care. By incorporating this podcast into the curriculum, nursing students are afforded the opportunity to refine their communication and relationship management skills, in alignment with the principles delineated in Annex A. Furthermore, the podcast facilitates the development of evidence-based competencies for supporting individuals of diverse age groups, encompassing an informed understanding of prevalent health conditions, including neurological conditions like PD.

In the context of the Parkinson’s podcast, the term “awareness” refers to a comprehensive understanding of the disease, including its clinical aspects, emotional impact, and the importance of compassionate care. The authors consider this multifaceted awareness crucial for nursing students, enabling them to deliver skilled and empathetic care across the entire range of PD, from pre-diagnosis to end-of-life. While specific conceptual frameworks for enhancing disease awareness among nurses don’t explicitly underpin this study, the podcast’s creation and evaluation align with established nursing pedagogical approaches. As noted, these approaches are based on the United Kingdom’s Nursing and Midwifery Council’s Standards of Proficiency for Registered Nurses, focusing on evidence-based practice, effective communication, and holistic patient-centred care, as detailed in Annex B. Thus, the podcast’s aim is to strengthen disease awareness in nursing students, fostering a more empathetic, knowledgeable, and capable nursing workforce to meet the complex needs of individuals with Parkinson’s Disease.

## Methods

### Study Aim

To assess the effectiveness of a co-designed Parkinson’s Awareness audio podcast as an educational tool for improving knowledge, awareness, and practice related to PD among nursing students in Northern Ireland.

### Objectives

1. To quantify the change in knowledge levels among nursing students regarding PD before and after listening to the co-designed audio podcast, as measured by pre-/post-test questionnaire scores.
2. To explore the impact of the audio podcast on nursing students’ perceptions and attitudes, specifically assessing changes in empathy and practice towards individuals with PD, through focus-group interviews conducted six months after exposure to the podcast.
3. To gather nursing students’ feedback on the podcast as a learning method, evaluating their perceptions of its effectiveness and usability as a tool for learning about PD.

### Design/setting/population

A mixed methods pre-test/post-test design was used to determine student knowledge and practice related to PD. The study was conducted using non-probability convenience sampling of year one undergraduate nursing students (n = 535) from Queen’s University Belfast (QUB) in Northern Ireland. All students were enrolled in their first year of nursing (including adult, children, mental health and learning disability fields of nursing) in Queen’s University Belfast were eligible for inclusion in this study.

### Co-design process

The development of the Parkinson’s awareness podcast followed a systematic and collaborative co-design approach consisting of several structured steps and mirroring best-practice in co-design methodology [25]. Each step contributed to the creation of a comprehensive educational podcast about PD.

**Step 1: Formation of the Co-Design Team** The co-design team comprised more than twenty individuals, representing a diverse range of perspectives. This team included people with lived experience of Parkinson’s Disease, dedicated carers, PD nurse specialists, nursing students, representatives from PD UK charity, and nurse lecturers from Queen’s University Belfast. This multidisciplinary team ensured a holistic approach to content development.

**Step 2: Co-Design Workshops and Content Creation** Early in the process, the co-design team conducted workshops involving these individuals, where they identified unmet needs and essential topics for the Parkinson’s awareness podcast. Thematic analysis of these workshops guided the initial structure and content of the podcast, ensuring it addressed the identified educational requirements.

**Step 3: Expert Advisory Team Involvement** The co-design team engaged with an expert advisory team consisting of individuals with diverse expertise, including academics, healthcare professionals, and charity representatives. These meetings focused on refining content and scripts for the podcast, aligning it with the specific needs of nursing education and Parkinson’s awareness.

**Step 4: Integration of Personal Experiences** To enhance the authenticity of the podcast, people with personal experiences of PD were involved in the development of the interview guides and scripts for podcasting. These people shared their insights and experiences across the illness journey, which were integrated into the podcast to provide relatable and valuable perspectives from the perspective of a person living with PD, a spouse (carer) of a person living with PD and a specialist nurse who provides care to people with PD.

**Step 5: Development of Prototype 1** The insights gathered from previous steps were synthesized to create a draft content structure. The podcast was designed as a 75-minute audio resource, organised into three main segments. These segments featured interviews conducted by nursing students with a person living with PD, a carer for someone with PD, and a PD specialist nurse. Scripting of these interviews was meticulously handled by the co-design team to ensure learning outcomes were met, while avoiding repetition.

**Step 6: User Testing and Refinement** An important phase involved user testing to evaluate the acceptability and effectiveness of the podcast among nursing students. This involved providing access to the podcast for a specified period and collecting feedback through pre/post-test questionnaires and voluntary focus group participation. The collected data from this user testing phase were analysed to identify areas for refinement, ensuring the podcast’s alignment with nursing education goals.

**Step 7: Development of Final Podcast** The feedback obtained from the user testing phase guided further refinements to the podcast content, structure, and presentation. These refinements were made in collaboration with the advisory group, ensuring that the final podcast met the educational needs of nursing students and effectively conveyed Parkinson’s Disease awareness.

### Intervention implementation

Year one nursing students were given access to the PD podcast for 30 days. Students were made aware that the podcast was an essential aspect of their year one nursing course. Students were permitted to listen to the podcast at any convenient time within this 30-day period and complete the voluntary pre/post questionnaire immediately before and after listening. The podcast was uploaded as an MP3 audio file within the Canvas Learning Management System used by year one nursing students and was not available for external download during this study. While participation in the podcast was a mandatory part of the programme, completion of the pre/post-test questionnaires and participation in focus groups was voluntary. A recording of the podcast can be found here: https://www.parkinsons.org.uk/professionals/podcast-learn-about-parkinsons.

### Data collection and recruitment

The data collection process for this study involved a multi-faceted approach to gather valuable insights from nursing students regarding their knowledge and perceptions of Parkinson’s Disease. Additionally, the recruitment strategy ensured that participants were provided with clear information and the freedom to participate according to their preferences.

All year one nursing students at Queen’s University Belfast were invited to participate in the study, resulting in a total eligible sample of 535 students. The recruitment process was initiated through email communication by a gatekeeper, not involved with this study, where students were informed about the ‘Parkinson’s awareness’ podcast, which was integrated into their year one nursing course. The email included a comprehensive information sheet, ensuring students had a complete understanding of the study’s objectives and requirements. Furthermore, contact details of the research team were provided, ensuring open lines of communication. To express their interest in participating, students selected the pre and post web links within the university’s Canvas Learning Management System, which granted them access to the evaluation questionnaires. Prior to completing each questionnaire, students were required to confirm their understanding of the participant information sheet and their voluntary consent to participate.

The data collection process included two main components: the administration of the Parkinson’s knowledge questionnaire and focus-group interviews, aimed at exploring the podcast’s impact on nursing students’ practice. The Parkinson’s knowledge questionnaire, consisting of 31 items, was based on previously published questionnaires [26, 27]. This instrument encompassed a range of statements, with the first 10 items derived from the work of Alwaleed et al. [27], addressing general aspects of Parkinson’s Disease. The subsequent 21 items, inspired by Bhidayasiri et al. [26], covered topics related to diagnosis, therapeutic treatment, and the disease course. Students were provided with links to complete the questionnaire before and after listening to the podcast.

In addition to the questionnaires, a co-designed interview guide was created to explore the podcast’s influence on student nursing practice during a six-month placement period following podcast exposure. Students were invited to self-register for focus group slots, providing flexibility in scheduling. These 30-minute focus groups were conducted online via MS Teams and were moderated by two members of the research team (GM & PS). The focus groups involved a total of 35 nursing students, organised into four groups, each comprising a maximum of eight participants. Topics discussed included overall impressions of the podcast and its role in improving and informing nursing practice.

Details of the questionnaire and interview guide can be found in supplementary files 1 and 2. Additionally, a copy of the consolidated criteria for reporting qualitative studies (COREQ) is available in supplementary file 3. It was made explicitly clear that participation in both questionnaires and focus groups was entirely optional and would not impact students’ module grades, ensuring their comfort and autonomy in the research process.

### Ethics

This study received ethical approval by Queen’s University Belfast’s Faculty of Medicine, Health and Life Sciences Research Ethics Committee (MHLS 20_143) in December 2020. The study took place between March 2021 and January 2022. All methods were performed in accordance with the Declaration of Helsinki.

### Consent

Each participant consented to complete questionnaires by ticking a box confirming they have read the information sheet and they consent to participate in this study. This form of consent was required for each questionnaire and participants were not able to begin either questionnaire until this option had been selected. In addition, each participant actively clicked on a link (from Canvas) before they could access the questionnaire. It was not possible to begin the questionnaire accidentally. With regards to the focus groups, participants interested in engaging had to register to attend, read the accompanying information sheet and provide written consent. Participants were also informed they were under no obligation to complete any of the questionnaires or attend focus groups. Participants were permitted to withdraw from the study at any stage, without giving any reason and information about these processes was provided within the study information sheet.

### Data analysis

Participants supplied their student number when completing each questionnaire. This allowed researchers to match the pre-test and post-test data sets to use dependent paired t-tests. This information was anonymised after the data was paired. All data was uploaded to SPSS for statistical analysis. The research team were the only individuals with access to the Microsoft Forms data. All data collected during this study complied with the General Data Protection Regulations (GDPR). Electronic data was stored within a password protected file on a university computer and in an anonymous format, identifiable only by unique participant identifier. Storage of data was in accordance with the GDPR (2018). The anonymised data will be retained for 5 years after study completion before being permanently deleted. The research team used descriptive statistics and dependent paired t-tests to analyse their results. The team did not collect any demographic details on participants during this evaluation as it was not deemed necessary.

The focus-group data was audio-recorded and fully transcribed verbatim by the research team. This data was then analysed for thematic content. This method of analysis facilitated familiarity with the data and facilitate the researcher to recognise themes as they emerged.

## Results

### Questionnaires

#### Analysis methodology

Raw total scores from the pre-test and post-test data were converted to percentage of correct answers in SPSS version 27. From this point, all analyses were conducted using the percentage-based scores. Descriptive statistics were calculated for pre-test and post-test scores. Distribution of pre-test scores was examined using a histogram to investigate any floor or ceiling effects or potential for regression to the mean when comparing with post-test scores. A paired t-test was conducted to examine the change from pre-test to post-test.

Raw scores for four of the five sub-scales (general knowledge of PD, diagnosis of PD, treatments for PD and trajectory of PD) were also converted to percentages in SPSS. A set of four paired comparisons using pre-test and post-test scores for these four subscales was conducted. The fifth sub-scale, symptom recognition of PD, was scored in a binary manner (0 vs. 10 points) for correct recognition of a list of symptoms. It, therefore, was analysed using a non-parametric binomial McNemar’s test (equivalent to a chi-square for testing differences between two categorical variables).

A Bonferroni correction was applied to the alpha value when determining the statistical significance of the results of these analyses, to reduce the risk of false positives associated with multiple comparisons (Altman & Bland, 1995). Alpha (0.05) was divided by the total number of comparisons in this study [6] to give an alpha value of *a* = 0.008. P-values, therefore, had to be below 0.008 to for the t-test result to be considered statistically significant. Analysis was conducted in a listwise manner, and all available data was used for pre-test and post-test descriptive statistics.

#### Missing data

Primary analysis was possible for N = 332 cases out of a total of 405 participants for whom data was recorded at either pre-test, post-test or both pre-test and post-test. If a participant was missing either pre-test or post-test, they were not able to be included in the primary analysis (Table 1).

**Table 1:** Missing data

	Missing n (%)
Pre-test data	25 (6.2%)
Post-test data	48 (11.9%)
Paired comparisons	73 (18%)

#### Primary analysis results

**Fig. 1:**
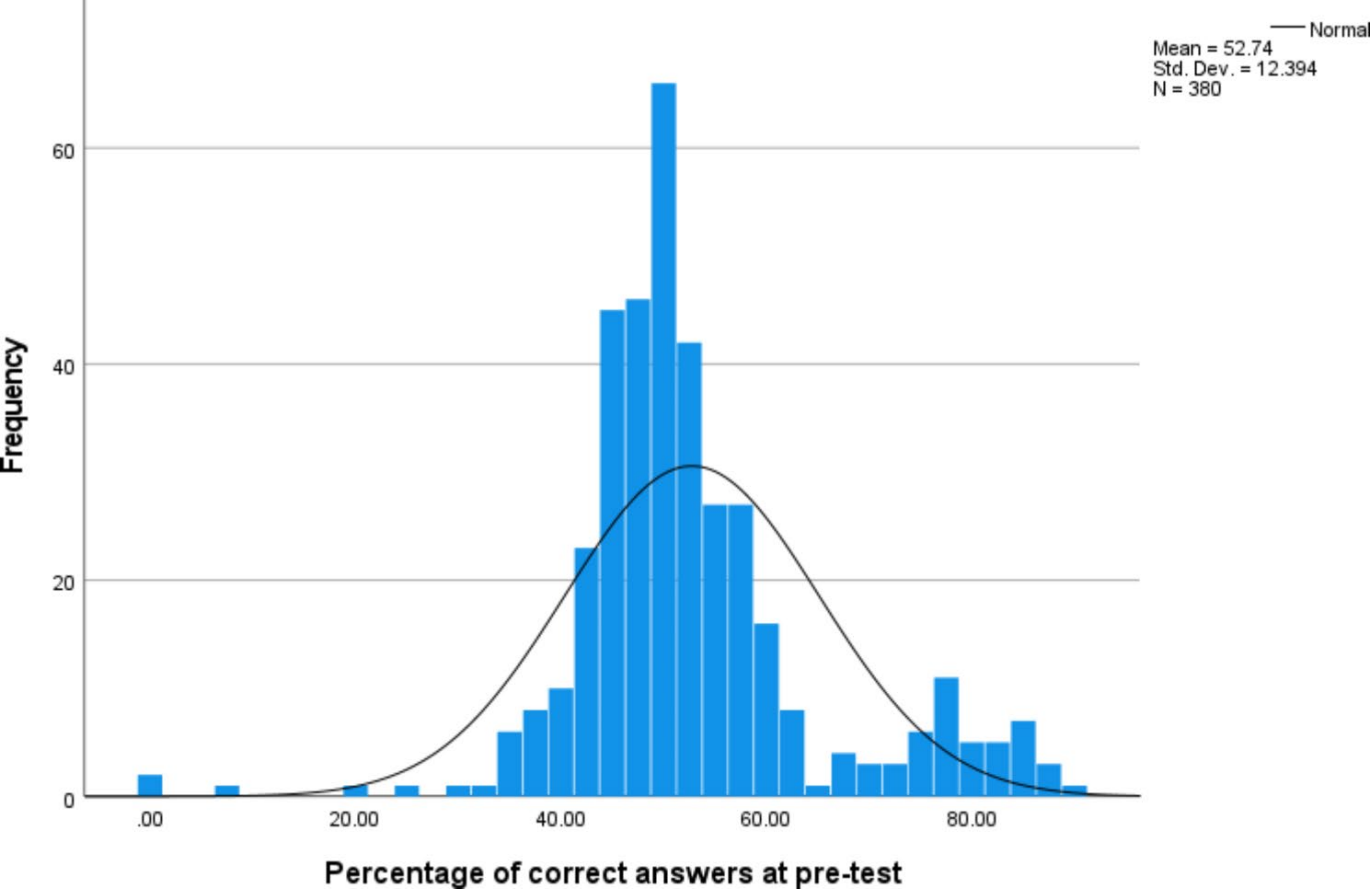
Distribution of pre-test scores

The distribution of pre-test scores (Fig. 1) shows that there is an approximately normal distribution, with no strong positive or negative skew and no evidence of ceiling or floor effects.

Descriptive statistics (Table 2) show that post-test scores (M = 80.07, SD = 17.32) were higher than pre-test scores (M = 52.74, SD = 12.39). This improvement from 52% mean score at pre-test to 80% mean score at post-test was statistically significant as indicated by the paired samples t-test which gave a result of t (332) = 26.68, p < 0.001.

**Table 2:** Descriptive statistics for pre-test and post-test total scores

	N	Range	Minimum	Maximum	Mean	Std. Deviation
Pre-test total score	380	90.00	0.00	90.00	52.7368	12.39374
Post-test total score	357	65.00	35.00	100.00	80.0700	17.31733
Valid N (listwise)	332					

#### Secondary analysis results

Descriptive statistics showed that general knowledge, diagnosis knowledge, treatment knowledge and illness trajectory knowledge sub-scale scores increased from pre-test to post-test (see Table 3). General knowledge increased by 7% points, diagnosis knowledge by 23% points, treatment knowledge by 17% points and disease trajectory knowledge by 21% points. All comparisons were statistically significant at the p < 0.001 level (see Table 4).

**Table 3:** Descriptive statistics for pre-test and post-test sub-scale scores

	N	Range	Minimum	Maximum	Mean	Std. Deviation
Pre-test general knowledge	380	100.00	0.00	100.00	82.8158	13.10472
Post-test general knowledge	357	60.00	40.00	100.00	90.4482	10.90411
Pre-test diagnosis	380	100.00	0.00	100.00	53.8947	17.98825
Post-test diagnosis	357	80.00	20.00	100.00	76.5826	22.19872
Pre-test treatments	380	100.00	0.00	100.00	61.1513	16.99488
Post-test treatments	357	75.00	25.00	100.00	78.2213	18.21921
Pre-test trajectory	380	100.00	0.00	100.00	56.9925	18.35925
Post-test trajectory	357	85.71	14.29	100.00	77.7911	20.00609
Valid N (listwise)	332					

**Table 4:** Paired comparisons of pre-test and post-test sub-scales

		Mean	Std. Deviation	t	df	p
Pair 1	Post-test and pre-test general knowledge	7.31928	14.51264	9.189	331	0.000
Pair 2	Post-test and pre-test diagnosis	22.77108	27.14238	15.286	331	0.000
Pair 3	Post-test and pre-test treatments	17.01807	23.30576	13.305	331	0.000
Pair 4	Post-test and pre-test trajectory	21.29948	24.69336	15.717	331	0.000

The analysis of the symptom recognition sub-scale showed that 62.3% of the sample improved from failing to recognise the list of symptoms at pre-test to recognising the list of symptoms at post-test, whereas only 25% of the sample remained incorrect from pre-test to post-test (see Table 5). This change was significant at the level of p < 0.001 according to a McNemar’s test.

**Table 5:** Change from pre-test to post-test on Symptom Recognition

			Pre-test		Total
			Incorrect	Correct	
Post-test	Incorrect	Count	83	1	84
		% of Total	25.0%	0.3%	25.3%
	Correct	Count	207	41	248
		% of Total	62.3%	12.3%	74.7%
Total		Count	290	42	332
		% of Total	87.3%	12.7%	100.0%

Overall, student nurse knowledge of PD was relatively low prior to listening to the podcast (M = 52.74, SD = 12.39). This increased significantly after listening to the podcast (M = 80.07, SD = 17.32). When this improvement was analysed as changes in specific types of PD disease knowledge, the most promising changes were found for diagnosis knowledge, disease trajectory and symptom recognition were found, and all changes were statistically significant.

#### Acceptability of the podcast

Participants were asked a series of items about their engagement with the podcast (Table 6). In response to both “The Parkinson’s Awareness podcast is a good learning resource” and “The Parkinson’s Awareness Podcast was straight-forward and easy to understand”, 95% of participants responded positively. In response to both “The Parkinson’s Awareness Podcast met my learning needs as a year one nursing student” and “I would recommend this Parkinson’s Awareness Podcast to others”, over 90% responded positively. Opinion about the length of the podcast showed more variability – 62% agreed that it was an appropriate length, but 13% of participants felt it was too long. There were also a notably higher proportion of neutral responses to this item (24%). In terms of listening to the podcast again in the future, 62% said they would listen again, with the remainder stating they would not. Overall, the response to the podcast content seems to be overwhelmingly positive, but length and potential for repeat engagement was less positive.

**Table 6:** Acceptability ratings of the podcast

		N	%
The Parkinson’s Awareness Podcast is a good learning resource	Strongly disagree	1	0.3%
	Disagree	0	0.0%
	Neutral	16	4.5%
	Agree	121	34.0%
	Strongly agree	218	61.2%
The Parkinson’s Awareness Podcast was straight-forward and easy to understand	Strongly disagree	2	0.6%
	Disagree	1	0.3%
	Neutral	13	3.7%
	Agree	127	35.7%
	Strongly agree	213	59.8%
The Parkinson’s Awareness Podcast met my learning needs as a year one nursing student	Strongly disagree	1	0.3%
	Disagree	3	0.8%
	Neutral	23	6.5%
	Agree	140	39.7%
	Strongly agree	186	52.7%
I would recommend this Parkinson’s Awareness Podcast to others	Strongly disagree	1	0.3%
	Disagree	2	0.6%
	Neutral	20	5.6%
	Agree	119	33.5%
	Strongly agree	213	60.0%
The Duration of the Parkinson’s Awareness Podcast was appropriate	Strongly disagree	0	0.0%
	Disagree - it was too long	45	12.7%
	Disagree - it was too short	1	0.3%
	Neutral	86	24.2%
	Agree	128	36.1%
	Strongly agree	95	26.8%
I will listen to this podcast more than once	No	116	32.6%
	Yes	240	67.4%

### Focus Groups

A series of focus groups were conducted six months after students listened to the original podcast. Following thematic analysis, three themes emerged in relation to effectiveness of a podcast as an educational tool to inform practice for nursing students. The first theme was that the podcast helped students to empathise and understand the lived experiences of people living with PD. The second theme was around how the podcast improved student care of people with PD, for example ‘getting medication on time’, the importance of PD nurse specialists and how to contact/refer patients to these services and how better support PD carers. The third theme focused on podcasting as an approach to education about PD. Supplementary 4 provides readers with the qualitative analysis code hierarchy.

### Theme one: fostering Empathy

The first theme that emerged from the data related to people living with PD and their experiences outside of a hospital or care setting. Participants in this study conceded that before listening, they often perceived people living with PD to be very unwell often requiring a lot of nursing and medical support. Participants identified that after hearing from a person living with PD, they understood how actively people with PD could live with a diagnosis.

*‘It struck me that people with Parkinson’s aren’t always so vulnerable, you know? I remember the man who was interviewed, and it struck me that this guy wasn’t what I expected. I mean, he wasn’t sitting in a nursing home all day or needing full-time care – he was living a life – making this podcast for one thing [laughs]. The lady carer was also excellent – she talked about going to rugby matches or to the pub with her husband who had Parkinson’s and that sort of just brought it home to me’.*

#### FG3P2

I liked hearing about the personal experiences of those living with Parkinson’s and how their families were coping with the diagnoses. It was also eye opening to see those with Parkinson’s still very well spoken and able to communicate well.

#### FG1P5

Most participants in this study also agreed that they had poor awareness about the challenges people living with PD encountered in their communities. Participants expressed how valuable it was to gain an insight from the person’s perspective by listening to the podcast, specifically in terms of enhancing their understanding of existing stereotypes that they or others might have.

*‘The bit about how people with PD are treated hit me. After listening, it is quite obvious they [the public] don’t know enough about this disease [PD]. Like, being mistaken for being drunk or not giving people enough time to pay money at a shop or things like that’.*

#### FG4P4

I remember listening to the podcast some months ago and while I can’t remember every aspect, the one part that I took away was that, whether in care or in society, you should take a bit more time and show a bit more patience when with people with PD.

#### FG2P7

After listening to the audio podcast, participants expressed increased understanding about living with PD in society. Participants also suggested that this increased understanding supported them and could support others to be more confident in communicating and supporting people with PD they encounter both in practice and in the general community.

*‘It sort of humanises the people with PD a bit more. I would get like quite scared of like talking and they wouldn’t really know how to talk and react to certain situations involving people with Parkinson’s and stuff, but this has given me confidence…just sort of remembering no, look you just talk to them and still-they’re still human at the end of the day. And it isn’t always about giving nursing care alone, so even if I am not working and I come across someone with PD, I feel more confident in knowing what challenges they have faced and how I might help, like helping people with buttons on coats or something’.*

#### FG1P4

I think that podcast can make you feel less overwhelmed and at least you’ll have a little bit of knowledge to kind of know what to do or what not to do at least.

#### FG1P3

Overall, this theme has illustrated that after listening to the podcast, nursing students interviewed report to have a greater awareness and appreciation of what living with PD was like outside of a hospital or care setting. Listening to the podcast, and in particular interviews with people with lived experience, reinforced that people with PD can live well in their local communities but there are significant challenges that can prohibit this. These experiences have the potential to foster empathy in nursing students who often are now aware of these challenges.

### Theme two: optimising practice

The second theme that emerged from the data related to how student nurses used their learning to improve their clinical practice when caring for people with PD in care settings. Participants articulated how providing individualised and holistic care could often be difficult in what they perceived to be fast-paced clinical areas with staffing shortages and time pressures [9, 10]. This is especially apparent during the Covid-19 pandemic where healthcare staff are facing even less face-to-face contact with patients and greater communication barriers existed through the wearing of PPE. Participants expressed how focussing on lived experiences of PD allowed them to step back and return to the basis of nursing practice with one participant commenting:

*‘I think it makes me have, you know better empathy skills, you know putting yourselves in their shoes and really realising what it’s like to live with Parkinson’s every day. Because we have an appreciation of this, it sort of will inform our nursing practice when they come into hospital or whatever, you know because we kind of are aware of the whole picture’.*

#### FG2P5

One aspect of the podcast discussed information surrounding medications for PD such as Levodopa. These medications are often referred to as ‘critical medications’, meaning they must be administered on time, according to the person’s unique schedule [26]. These medications act on the central nervous system to compensate for a deficiency of dopamine in the brains of those living with PD [26, 27]. This lack of dopamine causes the classic symptoms of PD including tremor, slowed movements, impaired posture and balance and speech changes. Collectively, participants agreed that the podcast introduced them to the importance of timely medication administration and the impact it has on patients’ symptoms and activities of daily living. One participant expressed:

I was on placement saying about his critical meds at 7 in the morning and I was just a bit like ‘Oh is it going to be making that much of a difference?’ But once you listen to the podcast it really does help you understand that.

#### FG1P6

*‘The point about medications really stuck with me. I remember being placed in a care home after and helping with medications and noting that a man with Parkinson’s had a 12pm dose of Levodopa and this was always given late – like 12:30 − 12:45pm because of lunches and whatever. So, I was able to say to the nurse about the podcast and she took it on board and seemed to prioritise it from then on’.*

#### FG3P4

While most nursing students discussed the administration of critical medications as an important part of practice, others also mentioned about their role in providing care that was truly tailored to someone with PD. Nursing students described how they often took a bit more time with people with PD, for example helping them to tie laces on their shoes, helping them button their shirts, supporting them to brush their teeth after receiving time critical medication and so on. Another example of this was supporting people with PD to mobilise and engage with exercise therapies.

Sometimes it would be a bit quieter in the afternoon [on the ward] and I would just go into John [Pseudonym – Patient with PD] and be like, right let’s go for a walk and get the legs moving or whatever. I remember on the podcast it being drummed into us that slowness and stiffness was a big challenge, and a bit of exercise could help.

#### FG1P5

The inclusion of an interview with a Parkinson’s nurse specialist was also beneficial for listeners. Many students articulated that they were not aware that such a role existed in the UK, nor was there awareness about the criticality of this role for people living with PD.

*‘I think the biggest thing I took was that actually there is an expert I can contact [a PD Nurse Specialist] and that expert has an office number and can do things like prescribe medications or provide a referral to a specialist. I didn’t know this service existed and I guess others might not either’.*

#### FG4, P4

*‘I really liked all of the podcast, the wee stories, the different speakers and so forth. The Parkinson’s nurse was really good. When I heard her speaking, I thought, Oh – I didn’t know you could things like provide advice, write prescriptions, arrange an urgent referral to the neuro doctor or whatever. I mean – this sounds like such an important role and when I was on my last placement, the staff didn’t seem to know about this person before I came onto the ward – but they do now and hopefully that helps’.*

#### FG3, P6

After listening to the PD podcast, nursing students reported how they were empowered to help make a positive impact on practice. Advocating for timely administration of critical medication, exercise routines and involvement of the PD nurse specialist were actions that appeared to arise as a result of listening to the audio podcast.

### Theme three: podcast acceptability

The first two themes considered people living with PD, both in the context of raising awareness and promoting understanding of lived experience and the action nursing students can take after listening. The third and final theme that emerged from the data related to audio podcasting as a method for providing acceptable education about PD. There was consensus amongst all people that podcasting was an excellent way to deliver education about PD as it incorporated the voice of a person living with PD, a carer of someone with PD and a PD nurse specialist. Some suggestions also emerged from the participants’ feedback to enhance the podcast’s impact. These include considering a shorter podcast duration to align with attention spans, providing explicit guidance on effective podcast engagement (such as note-taking or supplementary materials), exploring additional topics relevant to PD care (e.g., palliative care and treatment methods), and maintaining a high level of production quality.

Participants in the focus groups expressed their appreciation for the podcast’s comprehensive approach, highlighting its focus not only on individuals with Parkinson’s but also on their family members and carers. This multi-perspective approach provided a rich and diverse understanding of the condition, making the learning experience more valuable. Additionally, the inclusion of guest speakers was found to be highly beneficial, making the podcast relatable and illuminating the various ways PD can impact individuals while showcasing their coping strategies. These perspectives have contributed to a heightened awareness among nursing students about the complexities of the disease, fostering a deeper understanding of the challenges faced by those affected.

I liked that the podcast focused on individuals with Parkinson’s but also on their family members and their carers, I felt it gave a really good overview of the condition from different perspectives.

#### FG4P4

*‘It was good that there was guest speakers on the Podcast, it made it more relatable and I found it so interesting to see how PD can effect different people in different ways and how they have ways of coping with it. It has made me more aware of the disease’.*

#### FG3P2

While participants expressed that the highlight of the podcast was the lived experience of PD, there was some participants who felt that the podcast duration could be shorter.

Some participants suggested that they felt a podcast of 75 min was too long and had a negative effect on their motivation to keep listening. Most participants agreed that to maintain engagement in the content, it would be useful to break it up into multiple segments with one participant suggesting:

I would say about 40 min is an appropriate length, ‘cause I tend to lose- not lose interest but I kind of drift off a bit after about 15 and 20, so if it was like 40 minutes I could do it but in like two like sittings, so I say that would be a good time.

#### FG2P1

It was straight to the point however I believe the Podcast could have been shortened to 30 min or so.

#### FG1P1

In addition to the duration of the audio podcast, participants also agreed that there were differences in how learners used the podcast. Some participants noted how they would sit down with a notepad to take notes as they would in a traditional online lecture, while others engaged with the resource as though it was a radio interview and therefore did other activities while they were listening. Some participants suggested that there should be recommendations from the co-design team about how best to use the audio podcast and furthermore, provide recommendations for supplementary reading and educational videos for those that wanted to learn more after listening.

*‘This is going to sound negative, but it really isn’t, I just know our class struggled a wee [little] bit in relation to knowing what we were meant to do. Some girls [students on the programme] listened to it at the gym, some did it like a lecture and took notes and so on. So, I think just a bit of advice on what we should do to get the best out of it would be my recommendation’.*

#### FG3P3

I listened to the podcast twice, it was great. It didn’t tell me everything I needed – for example, I wanted to know more about deep brain stimulation as this was happening in my placement and this wasn’t covered in detail on the podcast. So, if there was like a video or reading that helps us after that would be great.

#### FG3P6

I expected the podcast to focus more on palliative care to be honest – we had a lecture a few weeks before the podcast and it was all about palliative care for everyone with incurable disease, like PD, and then we got to this podcast, and it seemed more about living well now and our role in helping quality of life. I think these could be brought closer together to be honest and maybe some sort of video or reading about this could help.

#### FG2P8

Participants discussed the production of the Parkinson’s podcast as being one of their favourite aspects, which included narration, background music and employed different segments. They also expressed that this was beneficial in recapturing their attention at different stages and helping them become more engaged when they became distracted with one participant summarising:

*‘Hearing different speakers, having different segments, the background music was really good. It felt like a professional podcast – you could tell the people who made it were professionals’.*

Whoever had the idea to have actual nursing students from Queen’s (QUB) lead the interviews on the podcast was brilliant. It really resonated with me, you know, like they are like me (as a nursing student) asking questions I might ask.

#### FG1P8

*‘It makes a difference you know, having a podcast that is professionally put together. It is a big turn-off for me to hear podcasts that have bad sound quality or aren’t edited or bits are left in that aren’t meant to be there and stuff. I’m used to listening to Joe Rogan or TalkSport or MMA and stuff and so my standards are quite high when it comes to podcasting’.*

#### FG2P6

Participants felt that this audio podcast was an invaluable tool for allowing the voices of people affected by PD to be heard. It is also a useful way to encourage those affected by PD to be involved in educating undergraduate nursing students on the challenges they face and what is most important to them. Consideration to podcast duration, availability of supplementary learning resources and quality of production were factors that students felt most contributed to the strength or limitations of using podcasts in higher education.

## Discussion

Audio podcasts have made finding and listening to information educational, entertaining and easily accessible [28]. It allows experts to share knowledge with a wide range of listeners, and its pre-recorded nature means it is available at any time to listeners [28]. This method caters to students with varying study habits and allows students to learn at their own pace and engage in other activities while listening [29]. A recent study also suggests podcasts may result in increased knowledge retention when compared to reading a book Chap. [30]. Despite the use of podcasts in healthcare education being an increasingly popular resource, there is limited guidance about effectively developing and evaluating podcasts [31, 32]. The primary aim of this study was to implement an educational resource which facilitates students in developing greater knowledge and understanding about PD.

Not only has lack of public awareness of PD been made apparent, but recent studies have also highlighted poor knowledge among healthcare professionals including nurses, doctors and physical therapists [33–36]. This is important in the context of the study as student nurses also have an important role to play in the care of people with PD. They are seen as role models for the public, likely influencing policy and teaching others about the disease. Because of this, student nurses are required to have good understanding of PD to disseminate their knowledge and demonstrate best practice. By raising awareness of the disease, student nurses will develop an increased understanding, enabling them to provide support, monitor symptom progression and promote person-centredness to those living with PD.

In this study, students favourably considered podcasts as a useful educational tool to support their learning about PD. They found it to be beneficial for applying knowledge gained from the podcast to their past experiences in healthcare, both personal and professional, and found it valuable for their career moving forward. Students also note how gaining the perspective from people affected by PD was an invaluable aspect of the podcast, giving a personal insight into what it is like living with or caring for someone with the disease. Tevendale and Armstrong [37] discuss how stories from patients and their families can enable students to develop a deeper understanding of specific experiences, encourage problem solving and identify what it most important to service users.

The findings of the study confirmed an increase in knowledge of PD post-podcast across all questionnaire categories. Post-test results show a greater knowledge and understanding of PD compared with pre-test in all 31 items post-podcast, specifically with regards to knowledge, diagnosis, treatment and trajectory of PD. Students felt the podcast was helpful in overcoming stereotypes and resolved previous misconceptions about PD. They also discussed how it gave them confidence in caring for people with PD and was successful in positively influencing their clinical practice. The co-design methodology that was employed in the development of this audio podcast is also likely to be a contributing factor to the success of this educational intervention. Co-design methodology has been consistently associated with positive outcomes in as it pertains to healthcare education as it facilitates learning developers to appropriately integrate the voice of experts with lived experience [38–43].

There is a paucity of literature surrounding PD education for healthcare professionals in general, but more specifically in relation to education for undergraduate student nurses. This lack of education for undergraduate nurses on PD is a cause for concern as without this, students are unable to fully understand the condition and provide a high standard of care for those living with PD. The present study illustrated the impact of an audio podcast for delivering undergraduate nursing education for PD.

### Strengths, Limitations and Recommendations

To our knowledge, this study is novel as it has investigated the impact of a PD awareness podcast for year one undergraduate nursing students. Dissemination of the podcast resulted in significant improvements in knowledge and understanding of PD amongst undergraduate student nurses’ post-intervention. This in turn, has the potential to result in better care for those with PD and greater dissemination of knowledge and best practice. A co-design methodology was used to capture lived experience of those affected by PD to develop the audio podcast and this learning resource was evaluated across many participants (n = 332), finding significance. Although the results are representative of a large cohort, the sample was from one university in Northern Ireland, possibly limiting the generalisability of the findings. Demographic data was not collected from nursing students which may have determined previous care experience or knowledge of PD that would be relevant to the study. Further, the conduction of focus groups using an online tool is likely to lead to different dynamics among participants when compared to face-to-face data collection. Another potential limitation of this study is that it was not pre-registered (e.g., ISRCTN Registry or Open Science Framework), which could potentially introduce concerns about publication bias or selective reporting. However, it’s important to note that this study did not involve a randomised controlled trial (RCT) or an experimental design. In the context of education research, such designs are less common, and the primary focus was on evaluating the impact of a co-designed Parkinson’s Awareness audio podcast among nursing students in Northern Ireland. The absence of pre-registration, while a recognised limitation, is balanced by the study’s focus on a unique educational intervention and its mixed methods evaluation approach.

The implementation of PD educational interventions across multiple universities or multiple groups of healthcare professionals would be a recommendation for future research. Further follow-up studies are also recommended to determine the extent to which nursing students retain their knowledge about PD and how it influences practice.

## Conclusion

Podcasts are increasingly being recognised as an effective platform to facilitate the education of undergraduate nursing students. Using podcasting as an educational resource has many benefits including ease of production, feasibility for listeners and ability to disseminate data to a wide audience. Despite this, there is a paucity of literature available on the effective evaluation of using podcasts for healthcare education, as well as limited evidence of PD education for undergraduate student nurses.

This study highlights how podcasting as an educational resource for undergraduate student nurses is a successful way of developing knowledge of PD. The lack of empirical research on innovative approaches to PD education is surprising due to the increasing prevalence of the disease. This study may act as a driving force towards the development of innovative approaches to healthcare education, specifically in relation to PD, enabling nursing students to recognise, assess and manage PD care effectively.

### Electronic supplementary material

Below is the link to the electronic supplementary material.

Supplementary Material 1

Supplementary Material 2

Supplementary Material 3

Supplementary Material 4

## Data Availability

The datasets used and/or analysed during the current study are available from the corresponding author on reasonable request.
