# Supplementary material for: Evaluation of a co-designed Parkinson’s awareness audio podcast for undergraduate nursing students in Northern Ireland
Source: BMC Nurs. 2023 Oct 9;22:370. doi: 10.1186/s12912-023-01544-x (PMC10561504; doi:10.1186/s12912-023-01544-x)
Supplement: Supplementary file 1 — Supplementary Material 1 [file 12912_2023_1544_MOESM1_ESM.docx]

**Supplementary File 1**: **Study Questionnaire**

**Evaluation of a Co-Designed Parkinson’s Awareness Audio Podcast for Undergraduate Nursing Students in Northern Ireland**

**Parkinson’s Knowledge Questionnaire (Alwaleed et al. 2018; Bhidayasiri et al. 2014)**

**Respondents to answer either True or False.**

1.Parkinson's Disease and Alzheimer's Disease are different names for the same disease

(1 Point)

True

False

2.Parkinson's Disease is a degenerative disease of the brain (associated with loss of brain cells)

(1 Point)

True

False

3.In Parkinson’s disease, the level of a chemical (neurotransmitter) in the brain called dopamine is reduced

(1 Point)

True

False

4.All patients with Parkinson’s disease experience tremor (involuntary shaking movements)

(1 Point)

True

False

5.Parkinson’s disease is more common in older individuals

(1 Point)

True

False

6.Parkinson’s disease can also affect young adults

(1 Point)

True

False

7. Parkinson’s disease usually affects multiple members of the same family

(1 Point)

True

False

8.There are new treatments that can cure Parkinson’s disease

(1 Point)

True

False

9.There are treatments that can improve the symptoms of Parkinson’s disease

(1 Point)

True

False

10.Patients with Parkinson’s disease often feel socially isolated

(1 Point)

True

False

11.Which of the following are possible symptoms of Parkinson's Disease (please select all that apply)

(1 point))

Tremors

Slowness of Movement

Imbalance

Rigidity

Memory Problems

Depression

Weight-Loss

Urinary Urgency

Insomnia (Difficulty Sleeping)

Pain

Excessive Daytime Sleepiness

Visual Hallucinations

Reduced Sense of Smell

Constipation

12.Definite diagnosis of Parkinson's Disease requires neuro-imagining confirmation

(1 Point)

True

False

13.Parkinson's Disease only affects patients after 55 years of age

(1 Point)

True

False

14.The presence of a rest tremor is mandatory for diagnosis of Parkinson's Disease

(1 Point)

True

False

15.The presence of bradykinesia (slowness of movement) is mandatory for the diagnosis of Parkinson's Disease

(1 Point)

True

False

16.Both smoking and high caffeine consumption increase risk of Parkinson's Disease

(1 Point)

True

False

17.Parkinson's Disease diagnosis can be confirmed by the response to a dopaminergic medication (e.g. giving Levodopa to a person with symptoms prior to formal diagnosis)

(1 Point)

True

False

18.Almost all patients diagnosed with PD should begin dopaminergic drug (e.g. Levodopa) as soon as possible.

(1 Point)

True

False

19.Most of the available drugs for Parkinson's Disease treat the symptoms of the condition AND slow the progression of the disorder

(1 Point)

True

False

20.The most effective drug available for Parkinson's Disease is usually Levodopa

(1 Point)

True

False

21.Levodopa has been shown to accelerate the progression of Parkinson's Disease

(1 Point)

True

False

22.Dopamine Agonists (e.g. Pramipexole or Ropinirole) have less risk of motor complications than Levodopa (e.g. Sinemet or Madopar)

(1 Point)

True

False

23.Dopamine agonists (e.g. Pramipexole or Ropinirole) have definite evidence on slowing disease progression

(1 Point)

True

False

24.Parkinson's Disease can be cured by deep brain stimulation surgery

(1 Point)

True

False

25.Patients with tremor predominant symptoms progress more rapidly in the degenerative process than those with postural instability and gait problems

(1 Point)

True

False

26.Becoming immobile or wheel-chair/bed-bound is inevitable in Parkinson's Disease

(1 Point)

True

False

27.Depression in Parkinson's Disease is partly caused by Dopamine deficiency

(1 Point)

True

False

28.The risk of dementia is greater for those patients with Parkinson's Disease

(1 Point)

True

False

29.Deep brain stimulation surgery can stop progression of Parkinson's Disease

(1 Point)

True

False

30.Current evidence supports the use of stem cell transplantation as a curative treatment of Parkinson's Disease

(1 Point)

True

False

31.Parkinson's Disease is a condition requiring palliative care

(1 Point)

True or False
