# Supplementary material for: Evaluation of a co-designed Parkinson’s awareness audio podcast for undergraduate nursing students in Northern Ireland
Source: BMC Nurs. 2023 Oct 9;22:370. doi: 10.1186/s12912-023-01544-x (PMC10561504; doi:10.1186/s12912-023-01544-x)
Supplement: Supplementary file 2 — Supplementary Material 2 [file 12912_2023_1544_MOESM2_ESM.docx]

**Supplementary File 2**: **Focus Group Interview Guide**

**Evaluation of a Co-Designed Parkinson’s Awareness Audio Podcast for Undergraduate Nursing Students in Northern Ireland**

**[All focus-group interviews moderated by two members of the research team]**

The purpose of this focus-group interview is to discuss how the podcast helped inform student nursing practice during placements. There are a few areas I would like to discuss about your experience. If any participant wishes to stop or withdraw from the interview at any time, please let me know and the interview will be stopped.

Topic Areas:

- What were your overall impressions of the podcast?
- What did you enjoy most about the podcast?
- What did you enjoy least about the podcast?
- Did this podcast influence or change how you provided care?
- Did you feel more confident about Parkinson’s Disease after listening?
- Did you feel more competent about providing care to someone with Parkinson’s Disease?
- Did the podcast help you to improve your practice?
- Did you use learning to help patients/service users, family members or colleagues overcome any problems?
- Did you work independently, or as part of a team, to provide innovative care or participate in quality improvement because of your learning?
- Did you modify your approach in your role as student nurse due to this podcast?
- Did you support colleagues on placement to adapt their practice in any way as a result of your learning?
- What are the limitations of the podcast?
- How might the podcast be improved for your colleagues in the future?
- What were the challenges of applying your learning from the podcast in practice?
- Did the podcast support you in all aspects of your role?

Thank you for your time, this has been very helpful, and I would be extremely interested in any other thoughts or feelings you have and would like to share to help me better understand your experience or is there anything you would like me to go back to?
