# Supplementary material for: Evaluation of a co-designed Parkinson’s awareness audio podcast for undergraduate nursing students in Northern Ireland
Source: BMC Nurs. 2023 Oct 9;22:370. doi: 10.1186/s12912-023-01544-x (PMC10561504; doi:10.1186/s12912-023-01544-x)
Supplement: Supplementary file 3 — Supplementary Material 3 [file 12912_2023_1544_MOESM3_ESM.docx]

**Supplementary File 4**

**Qualitative Analysis Code Hierarchy: Themes, Generic Codes, and Sub-Codes**

**Theme One: Fostering Empathy**

- **Generic Code 1:** Understanding Lived Experiences
  - **Sub-Code 1.1:** Perception of People with PD
    - *Generic Code 1.1.1:* Preconceived Notions
    - *Generic Code 1.1.2:* Realisation of Active Living
  - **Sub-Code 1.2:** Empathy and Perspective Shift
    - *Generic Code 1.2.1:* Empathetic Connections
    - *Generic Code 1.2.2:* Improved Patient Interaction
  - **Sub-Code 1.3:** Challenging Stereotypes
    - *Generic Code 1.3.1:* Overcoming Stereotypical Views
    - *Generic Code 1.3.2:* Impact on Nursing Practice

**Theme Two: Optimising Practice**

- **Generic Code 2:** Providing Quality Care
  - **Sub-Code 2.1:** Medication Management
    - *Generic Code 2.1.1:* Importance of Timely Medication
    - *Generic Code 2.1.2:* Criticality of Levodopa
  - **Sub-Code 2.2:** Individualized Care
    - *Generic Code 2.2.1:* Patient-Centred Approach
    - *Generic Code 2.2.2:* Addressing Specific Needs
  - **Sub-Code 2.3:** Impact of COVID-19 on Care
    - *Generic Code 2.3.1:* Challenges in Pandemic in Care Settings
    - *Generic Code 2.3.2:* Adaptation in Patient Interaction
  - **Sub-Code 2.4:** Role of PD Nurse Specialist
    - *Generic Code 2.4.1:* Awareness of Specialist Role
    - *Generic Code 2.4.2:* Collaborative Care Efforts

**Theme Three: Podcast Acceptability**

- **Generic Code 3:** Educational Podcast Evaluation
  - **Sub-Code 3.1:** Engagement and Length
    - *Generic Code 3.1.1:* Optimal Podcast Duration
    - *Generic Code 3.1.2:* Maintaining Listener Interest
  - **Sub-Code 3.2:** Student Learning Approaches
    - *Generic Code 3.2.1:* Diverse Listening Methods
    - *Generic Code 3.2.2:* Recommendations for Active Learning
  - **Sub-Code 3.3:** Supplemental Resources
    - *Generic Code 3.3.1:* Post-Podcast Learning Materials
    - *Generic Code 3.3.2:* Addressing Uncovered Topics
  - **Sub-Code 3.4:** Production Quality
    - *Generic Code 3.4.1:* Professional Presentation
    - *Generic Code 3.4.2:* Engaging Audio Elements
  - **Sub-Code 3.5:** Empowering Voices of PD Community
    - *Generic Code 3.5.1:* Inclusion and Representation
    - *Generic Code 3.5.2:* Impact on Awareness
