# Supplementary material for: Evaluation of a co-designed Parkinson’s awareness audio podcast for undergraduate nursing students in Northern Ireland
Source: BMC Nurs. 2023 Oct 9;22:370. doi: 10.1186/s12912-023-01544-x (PMC10561504; doi:10.1186/s12912-023-01544-x)
Supplement: Supplementary file 4 — Supplementary Material 4 [file 12912_2023_1544_MOESM4_ESM.docx]

**Supplementary File 3**

Consolidated criteria for reporting qualitative studies (COREQ): 32-item checklist.

| **No** | **Item** | **Guide questions/description** |
| --- | --- | --- |
| **Domain 1: Research team and reflexivity** |  |  |
| Personal Characteristics |  |  |
| 1. | Interviewer/facilitator | All interviews will be facilitated by both Dr Gary Mitchell (GM) and Dr Patrick Stark (PS). |
| 2. | Credentials | GM & PS both have a PhD and have expertise in qualitative research methods and qualitative data collection (including semi-structured interviewing and focus-group interviewing). |
| 3. | Occupation | GM is a Reader at Queen’s University Belfast in Northern Ireland. PS is a Lecturer at Queen’s University Belfast. |
| 4. | Gender | GM & PS are male. |
| 5. | Experience and training | GM is a registered nurse with considerable experience in higher education and care of older people. PS is a health psychologist and educationalist with considerable experience in higher education and care of older people. |
| Relationship with participants |  |  |
| 6. | Relationship established | GM & PS are lecturers are Queen’s University Belfast and teach on the Undergraduate Nursing Programme where students were recruited. |
| 7. | Participant knowledge of the interviewer | Participants would have been aware of who was conducting the focus groups as these were detailed in the information sheet and consent forms. |
| 8. | Interviewer characteristics | GM is a registered nurse with clinical expertise in the topic area. GM also has a strong awareness in the context (e.g., design, implementation and evaluation of podcasts in higher education). PS shares the same expertise and has collaborated with GM on similar research. |
| **Domain 2: study design** |  |  |
| Theoretical framework |  |  |
| 9. | Methodological orientation and Theory | Mixed methods evaluation comprised of pre/post-test questionnaire followed by thematic analysis of focus group data. |
| Participant selection |  |  |
| 10. | Sampling | Convenience sampling of 535 year one nursing students at Queen’s University Belfast undertaking BSc Professional Nursing Degree. |
| 11. | Method of approach | Participants were approached by a gatekeeper (Director of Education) that was not associated with the study. |
| 12. | Sample size | 332 participants in phase one and 35 participants in phase two (qualitative aspect). |
| 13. | Non-participation | Participants were reminded that participating in this research would not affect their course grade. This was noted in the information sheet and within the consent form. |
| Setting |  |  |
| 14. | Setting of data collection | Focus group data was collected via online meetings (MS Teams). Data collection took place during the student’s own time. |
| 15. | Presence of non-participants | There were no non-participants present during the focus group interviews. |
| 16. | Description of sample | Participants were year one undergraduate nursing students from Queen’s University Belfast. |
| Data collection |  |  |
| 17. | Interview guide | The interview guide was co-designed by people living with PD, carers of people with PD and academics involved in the study. The guide sought to explore how the podcast had influenced student nursing practice after listening. |
| 18. | Repeat interviews | No repeated interviews were carried out. |
| 19. | Audio/visual recording | All focus group data was audio-recorded. |
| 20. | Field notes | No field notes were collected during focus group interviews. |
| 21. | Duration | Focus group interviews lasted approximately 30 minutes. |
| 22. | Data saturation | Data saturation was achieved in this study with the participation of 35 nursing students in focus groups, wherein recurring themes and perspectives relevant to the podcast's impact on Parkinson's Disease awareness were consistently observed, indicating comprehensive exploration of the topic. |
| 23. | Transcripts returned | All participants had the option of reviewing their focus group transcript as noted in the information sheet, consent form and at the conclusion of the focus group interview. No participant requested this. |
| **Domain 3: analysis and findings** |  |  |
| Data analysis |  |  |
| 24. | Number of data coders | Qualitative data analysis, using thematic analysis (Braun and Clarke), was carried out by GM, PS, SCo & SCr) |
| 25. | Description of the coding tree | All authors actively participated in the design, data collection, analysis, and interpretation of the study, ensuring comprehensive collaboration and shared contributions to the research process. |
| 26. | Derivation of themes | The authors used thematic analysis to analyse themes. This was led by GM, PS, SCo, SCr initially and involved all team members in the advanced stanges. |
| 27. | Software | The authors used NVivo Version 12 to manage the data. |
| 28. | Participant checking | No member checking was carried out. |
| Reporting |  |  |
| 29. | Quotations presented | Direct quotations were presented from participants while confidentiality was maintained. |
| 30. | Data and findings consistent | The authors have ensured there was concordance between the data and findings that are presented. All authors were involved. |
| 31. | Clarity of major themes | The authors have reported all major themes as identified in this research. |
| 32. | Clarity of minor themes | The authors were not required to report on any minor themes or deviant cases within their presentation of results as these did not emerge. |
